# Salience and Valence of Appearance in a Population with a Visible Difference of Appearance: Direct and Moderated Relationships with Self-Consciousness, Anxiety and Depression

**DOI:** 10.1371/journal.pone.0088435

**Published:** 2014-02-06

**Authors:** Timothy P. Moss, Victoria Lawson, Paul White, The Appearance Research Collaboration

**Affiliations:** 1 Centre for Appearance Research, Faculty of Health and Life Science, University of the West of England, Bristol, United Kingdom; 2 Department of Psychology, School of Health, BPP University, London, United Kingdom; 3 Applied Statistics Group, Faculty of Environment and Technology, University of the West of England, Bristol, United Kingdom; Research and Development Corporation, United States of America

## Abstract

Psychometric measures of appearance salience and valence, CARSAL and CARVAL, have been previously demonstrated to be key factors underpinning appearance related self-consciousness and negative affect in the general population. However, the extent to which the scales are appropriate for people with a visibly different appearance has not previously been reported. Neither has the moderating effect of appearance salience (CARSAL) on the relationship between appearance valence (CARVAL) and appearance self-consciousness, previously shown in a general population sample, been replicated with people who are visibly different. Twelve hundred and sixty five participants with a visible difference in either secondary care (n = 651) or the community (n = 614) provided data. Analysis confirmed the psychometric qualities of both CARSAL and CARVAL, and the conceptual independence of each scale. The scales also demonstrated independent and interdependent relationships with social anxiety and avoidance in relation to appearance, depression and anxiety. Appearance salience moderated the relationship with valence on these psychosocial measures. In summary, this paper corroborates the use of CARSAL and CARVAL with both visibly different and general adult populations for the measurement of appearance salience and valence.

## Introduction

The recent development of two psychometrically robust self-report measures of appearance valence and salience has increased the tools available to understand distinctive components of a person’s appearance-related self-concept [1]. The rationale driving the original development of the Centre for Appearance Research Salience scale (CARSAL) and Centre for Appearance Research Valence scale (CARVAL) was to develop measures that could assess two aspects of appearance schema, the emotional evaluation of the self in relation to appearance (valence) and the extent to which self-relevant appearance information is brought to consciousness (salience). Furthermore, it facilitated investigation into interaction of these variables in relation to appearance related self-consciousness and social avoidance. Moss and Rosser conceptualized appearance schema as the “cognitive representation of organized information about the self in relation to appearance, which includes emotional and informational content about appearance, which serves also to guide information processing about one’s appearance” [1]. CARSAL sought to operationalize the extent to which appearance and physical self is brought into conscious awareness as an aspect of the working self-concept; CARVAL operationalized the extent to which the respondent evaluates his/her appearance in a positive or negative way. Moss and Rosser demonstrated in a general population sample that valence was related to self-consciousness of appearance and that this was moderated by salience. Increased appearance salience was shown to exacerbate the impact of negative appearance valence on appearance self-consciousness and social avoidance.

CARSAL and CARVAL provide more focused and briefer measures of the specific constructs of valence and salience than were previously available [2]. However, the initial development of the two scales was undertaken in a general adult population, and the authors highlighted the need for testing and validation in a population that are living with a visible difference (for example, those with scarring, visible skin conditions, or appearance altering congenital conditions). In addition to demonstrating the wider utility of the CARSAL and CARVAL scales, evaluating the potential moderating relationship of salience on valence in this population could be beneficial in guiding interventions. The current paper replicates the original Moss and Rosser CARSAL/CARVAL validation study [1] but with a visibly different rather than general population sample. The sample was recruited from both secondary care hospital settings, and primary care community settings to provide a broader range of participant perspectives and maximize recruitment.

The previous validation of CARSAL and CARVAL used the Derriford Appearance Scale 24 (DAS24 [3] ) as a principle outcome measure. DAS24 is a widely used, psychometrically sound measure that has been shown to be an effective and sensitive measure of appearance related self-consciousness and social avoidance [4]. It is less known in some medical settings, however, and consequently established measures of anxiety and depression, the Hospital Anxiety and Depression Scale (HADS) were also included as outcome measures [5].

The aims of the current research were to:

- Evaluate the psychometric properties of two existing measures of appearance salience and valence in a sample of participants with a visibly different appearance.
- Evaluate the relationship of appearance valence to appearance related self-consciousness and social avoidance, and examine the potential moderating effect of appearance salience on this relationship.
- Evaluate the relationships of appearance valence to anxiety and depression, and examine the potential moderating effect of appearance salience on these relationships.

We hypothesized that there would be a positive correlation between appearance valence, appearance self-consciousness, anxiety and depression. Furthermore, for each of these outcome variables, we hypothesized that appearance salience would moderate the relationship with appearance valence such that increased salience would amplify the impact of appearance valence.

## Methods

### Ethics

The research was approved by both the National Research Ethics Service UK Research Ethics Committee, and the University of the West of England Research Ethics Committee. Written consent was obtained from all participants in advance of their participation, which included appropriate information to ensure informed consent, an assurance of anonymity, and the right to withdraw without penalty.

### Participants

Sample size was based on recommendations by Comrey and Lee on minimum sample size in factor analysis [6]. They indicated that more than 500 is very good, whilst 1000 or more observations is excellent. For the current study, increasing sample sizes beyond 1000 served to enhance power and provided the opportunity to obtain a wide sample over multiple clinical groupings.

Participants aged over 18 years old who self-identified as being visibly different and with fluency in written and spoken English were recruited from community and clinical settings. Six hundred and fourteen community participants were recruited through advertisements and general practice doctors’ surgeries, whilst 651 participants were recruited via secondary care outpatient clinics. The clinics included prosthetics, dermatology, ophthalmology, general plastics and burns, ear, nose and throat clinics (including cleft lip and palate), cancer (head and neck, skin) and laser treatment. Participants were recruited from locations across the United Kingdom (Bristol, London, Bradford, Sheffield and Warwick). In total, 1265 participants were recruited. The measures, CARSAL/CARVAL, DAS24 and HADS, were included as part of a wider Appearance Research Collaboration study [7] assessing adjustment to visible difference, funded by the Healing Foundation. Those who agreed to participate were given a questionnaire booklet to complete at their next outpatient appointment or mailed the booklet. Participants self-reported demographic and visible difference information. Visible difference data included the cause of disfigurement from one of 11 options, for example, trauma, congenital condition, disease. These responses were then coded as either observable visible difference when clothed or less observable visible difference when clothed. Results are reported in Table 1.

**Table 1:** Participant demographic and visible difference information.

Female	68.5%
Male	28%
Undisclosed sex	3.5%
Age: Mean (SD)	47.34 (16.72)
Ethnicity: White	80.9%
Ethnicity: Black African or Caribbean	2.7%
Ethnicity: Indian or Pakistani	7.3%
Ethnicity: Other	1.6%
Ethnicity undisclosed	7.4%
Married or living with partner	61.9%
Living alone	22.7%
Living with friends relatives	14.4%
Marital status undisclosed	0.9%
Recruited from community	48.5%
Recruited from clinical setting	51.5%
*Observable visible difference	53.2%
**Less observable visible difference	46.8%

*Scalp, forehead, ears, eyes, nose, mouth, neck, cheeks, hands) to indicate areas normally visible to others when clothed.

**Chest, breasts, abdomen, back, genitalia, shoulder, upper arm, forearm, hips, buttocks, thighs, knees, lower leg, feet.

### Measures

A detailed explanation of the original item pool generation is provided in by Moss and Rosser [1] and is therefore not repeated here.

Prior to testing the existing CARSAL and CARVAL items in a population with visible differences, the guidance of both clinical and user experts was sought to exclude any items that had the potential to cause distress to this specific population. Subsequently, based on this expert consensus, two items were removed (“I like the way I look” and “My appearance makes me feel attractive”) as they were considered as being potentially sensitive for people with a visible difference. For a summary of the final items included see Table 2.

**Table 2:** Component loading of items for valence and salience using EFA [Principal axis factoring with oblimin rotation].

Item	Component	
	Valence	Salience
The way I look makes me unattractive	·746	
The way I look makes me feel good about myself**	·.743	
I feel bad about my body and appearance	·741	
I am satisfied with my physical appearance**	·768	
My body and face look pretty much the way I would like**	·737	
I don’t like the way I look	·741	
In most situations, I find myself aware of the way my face and body look		·595
I am often aware of the way I look to other people		·.787
I often think about the impression that the appearance of my face and body make		·.847
I am usually conscious of my appearance		·763
For me, my appearance is an important part of who I am		·734

*Rotated component loadings of magnitude>·20 shown; ** Positive items reverse scored.

#### Convergent criterion validity measures

In addition to the CARSAL/CARVAL, the other outcome measures were the DAS24 and HADS.

The DAS24 is a 24 item version of the DAS59 measuring social anxiety and avoidance in relation to appearance. Total scores range from 11–96 with lower scores representing lower levels of social anxiety and social avoidance. The authors report high internal consistency, with Cronbach’s alpha coefficient α = .92 [8]. DAS24 has previously been shown to relate to the constructs under current examination in existing validation study of CARSAL/CARVAL [1].

HADS is a valid and reliable 14 item questionnaire assessing anxiety and depression for patients with physical health problems. There are two separate domains for each, with scores ranging from 0 to 21, with higher scores indicating greater levels of anxious or depressed mood. For HADS anxiety Cronbach’s alpha α = ·83 and for HADS depression α = .82 [9].

#### Test-retest reliability

CARSAL and CARVAL were retested at 9 months in a subset of 349 participants (mean age 49.2 (SD 15.36), 25.2% males, 73.4% females, 1.4% sex unknown). No other scales were administered at the retest stage.

## Results

### Psychometric Properties of the Salience and Valence Scales

Missing data was defined as completing less that 50% of the total number of items. Missing values were missing completely at random and exclusion of incomplete responses did not substantially alter the results, consequently results for the full data set of 1265 participants are reported.

Items for both the salience and valence scales were evaluated to determine if they had skewed distributions and whether they exhibited either floor or ceiling effects. It was not necessary to exclude any items at this stage.

### Internal Structure for the Salience and Valence Scales

Item-total analysis for the final items in the salience scale demonstrated Pearson’s *r* correlations between .48 and .81, and internal reliability (cronbach’s alpha) α = .87. For the valence scale item-total analysis for the final items demonstrated Pearson’s *r* correlations between .67 and .7, and internal reliability (cronbach’s alpha) α = .88.

### Confirmation of Construct Identities

Analysis using Chatterjee’s statistic [10] indicates that there are no potentially influential multivariate observations which could otherwise grossly affect estimation of eigenvalues, factor loadings, factor scores, or the factors themselves. Kaiser-Meyer-Olkin measure of sampling adequacy indicated a sufficient sample for an exploratory factor analysis (KMO = 0·89), and Bartlett’s test of sphericity confirms a non-null correlation structure within the data (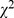 = 7207.2, *df* = 44, *p*<.001).

In a principal component analysis of total variance, an analysis using the bootstrapped Kaiser-Guttman criterion of retaining components with an associated eigenvalue greater than one [11] suggests a two component solution. Specifically, the eigenvalue and the associated 95 percentile bootstrap confidence interval for the first eigenvalue is 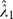 = 4.915 [95% CI 4.692 to 5.174], second eigenvalue 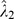 = 2.321 [95% CI 2·142, 2·507], and third eigenvalue 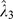 = 0·776 [95% CI 0.692 to 0.874]. Velicer’s Minimum Average Partial (MAP) correlation rule is widely used to determine the number of real factors and is based on the Exploratory Factor Analysis (EFA) concept of common factors. Analysis using Velicer’s MAP test [12] using the smallest average squared partial correlation coefficient and analysis using the revised MAP test on the smallest average fourth power partial correlation (see [13]) both identify a two component solution. Horn’s parallel analysis (PA) [14], using the 95 percentile limits is known to have good properties for the empirical determination of the number of factors both in PCA analyses and in EFA (see for instance, [15], [16]). Application of PA to a PCA extraction and to an EFA Principal Axis Factor Analysis indicates a two component or two factor solution.

**Figure 1:**
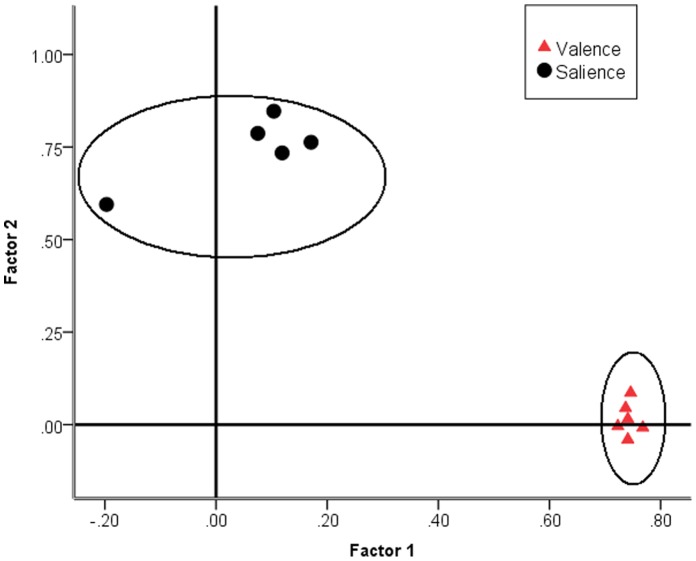
Component loading plot of items for valence and salience. Component loading plot of items for valence and salience using EFA [principal axis factoring with oblimin rotation].

Table 2 summarises component loading of items for valence and salience in a two factor EFA using principal factor analysis with oblimin rotation with parameter delta set to zero. A non-orthogonal rotation was used to allow correlation between factors and not otherwise force an orthogonal relationship. The factors are clearly differentiated with high loading of valence items on the valence construct but not on salience. A similar reverse pattern was observed for salience, with high factor loading on the salience but not the valence construct (see Figure 1). This pattern structure without any cross-loading was replicated using other non-orthogonal rotations (e.g. quartimax, equamax, promax) and using other factor extraction procedures (e.g. Maximum Likelihood). This robustness might be expected when there is a clear factor structure with strong factor loadings, and with a large ratio of sample size to number of parameters estimated. It is also noticeable that this factor structure replicates the factor structured reported by Moss and Rosser [1] in a general population (i.e. structural replication where same items load on a construct).

A main thrust of the current research is to determine the extent of similarity of the CARVAL-CARSAL factor structure observed in the general population and in a visibly different population. For these purposes the principal axis factor solution from the general population may be compared with a procrustean rotated principal axis factor solution using Tucker’s Coefficients of Congruence. Lorenzo-Seva and ten Berge consider this procedure and write that a value of congruence coefficients “*in the range.85 to.94 corresponds to a fair similarity, while a value higher than.95 implies that the two factors or components compared can be considered equal*” [17]. For the valence factor Tucker’s coefficient of congruence was found to be CC1 = .98, and for the salience factor CC2 = .96.

### Regression Analysis

**Figure 2:**
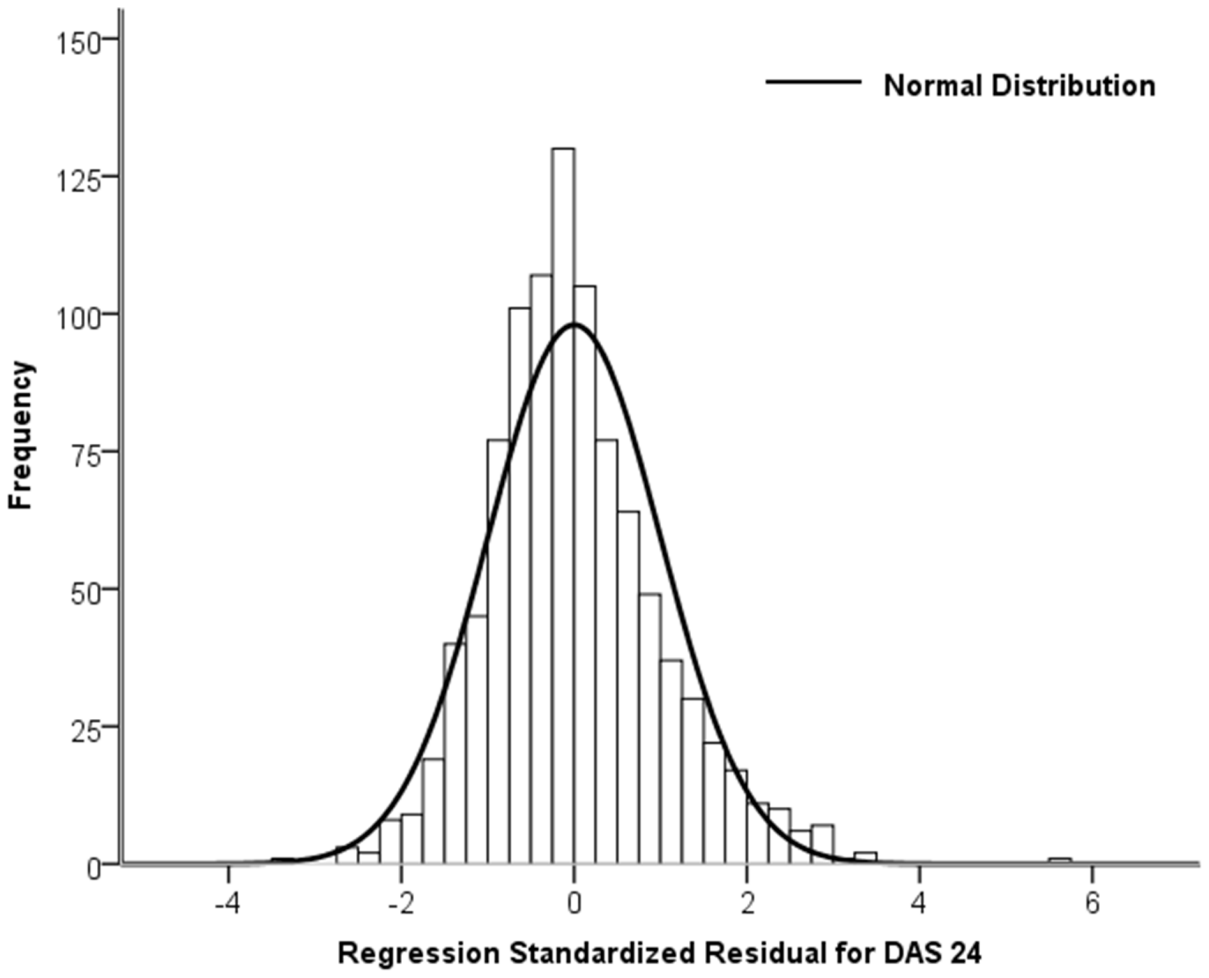
Distribution for CARSAL/CARVAL with DAS24 as the dependent variable. Distribution of standardized residuals for CARSAL/CARVAL with DAS24 as the dependent variable.

Multiple regression analysis was conducted with DAS24 as the dependent variable; CARSAL and CARVAL were entered as independent variables. A plot of the standardised residuals for the regression was approximately normal, justifying use of regression in this context, see Figure 2.

The overall model significantly predicted DAS24 score, adjusted *R* ^2^ = 0.469, F (2,975) = 289.78, p<.001. Both predictors provided significant independent contributions, CARVAL (*B* = .506, *t*(976) = 19.51, *p*<0.001) and CARSAL (*B* = .285, *t*(976) = 11.302, *p*<0.001).

Similar regressions were conducted for HADS anxiety and HADS depression, with CARSAL and CARVAL again entered as the independent variables. Plots of the standardized residuals for the both of these regressions were also approximately normal, and again justified the use of regression.

A similar pattern of interaction and main effects emerged. For anxiety, the overall model was significant, adjusted *R* ^2^ = 0.232, F (2,1192) = 119.6, p<.001. Both predictors provided significant independent contributions, CARVAL (B = .32, *t*(1189) = 11.3, *p*<0.001) and CARSAL (B = .25, *t*(1189) = 8.828, *p*<0.001. For depression, the overall model was significant, adjusted *R* ^2^ = 0.217, (F (2,1192) = 111.5, p<.001. CARVAL provided a significant independent contribution, (B = .42, *t*(1189) = 14.636, *p*<0.001), as did CARSAL (B = .058, *t*(1189) = 2.042, *p*<0.041). This smaller effect between salience and depression was expected given that salience of appearance does not necessarily equate to negative affect unless mediated by a degree of negative appearance evaluation. An analysis of variance inflation factors for each of the variables in all three of the regression models demonstrated no evidence of multicollinearity.

It was predicted that in addition to the independent effects of the scale, salience would moderate the relationship between negative valence, appearance self-consciousness and social avoidance (DAS24), anxiety (HADS anxiety) and depression (HADS depression). Specifically, poorer adjustment and increased levels of anxiety and depression would be predicted when high valence interacted with high salience.

**Figure 3:**
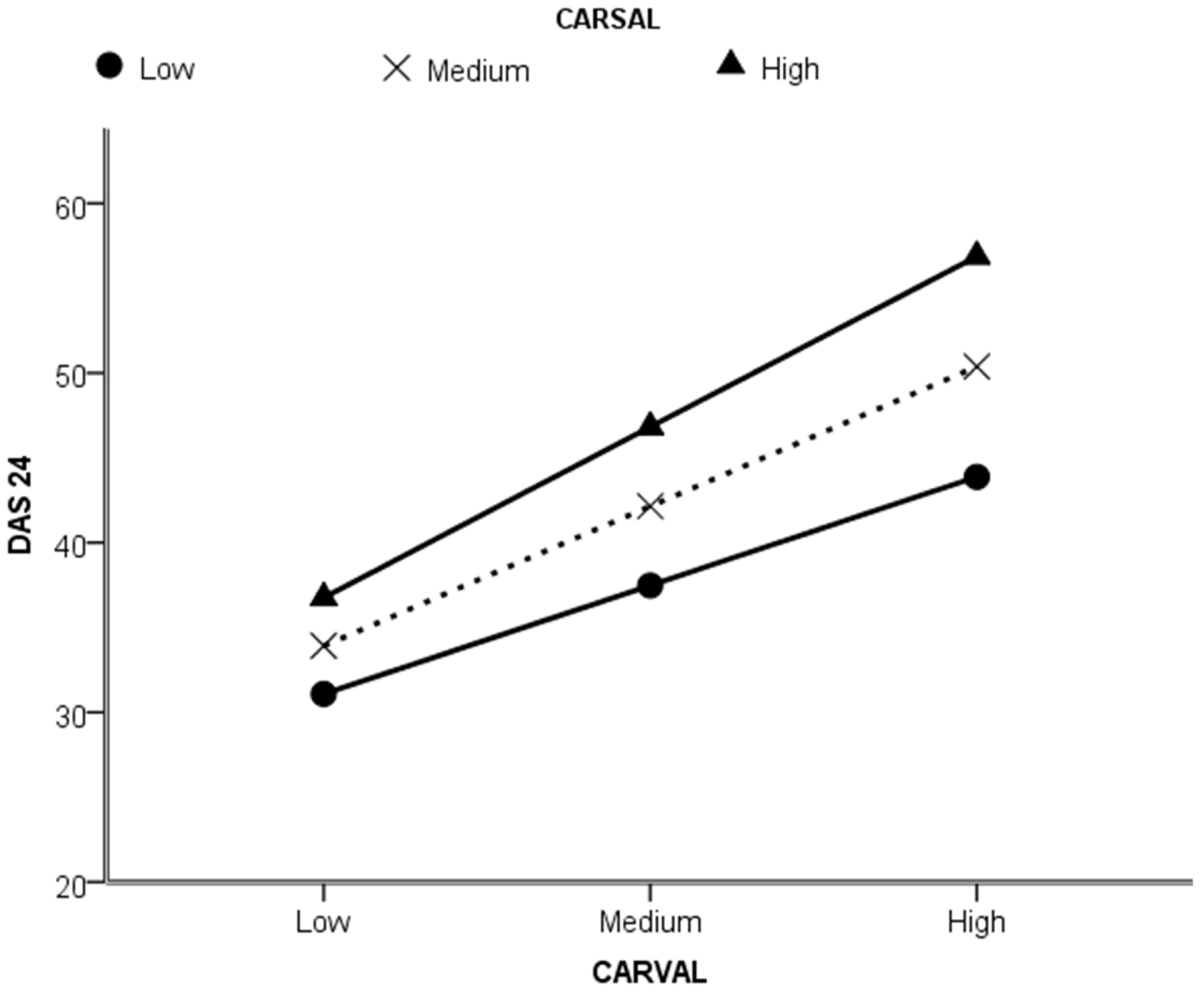
Moderation of CARVAL on DAS24 by CARSAL. Appearance self-consciousness and appearance valence relationship, based on continuous data split by low appearance salience (lower line), moderate appearance salience and high appearance salience (upper line). Graph produced on basis of continuous data by ModGraph-I (19).

To examine this, an interaction (moderation) term was calculated by multiplying the centred CARSAL and CARVAL scores and entering as a second step in the model along with centred CARSAL and CARVAL scores again, following entering centred CARVAL and CARSAL scores separately in step one [18]. The predicted interaction between CARVAL and CARSAL was also observed (B = 0.115, *t*(1189) = 4.701, *p*<0.001). As can be seen in Figure 3, higher DAS scores were associated with high negative valence and this was exacerbated by higher level of appearance salience.

**Figure 4:**
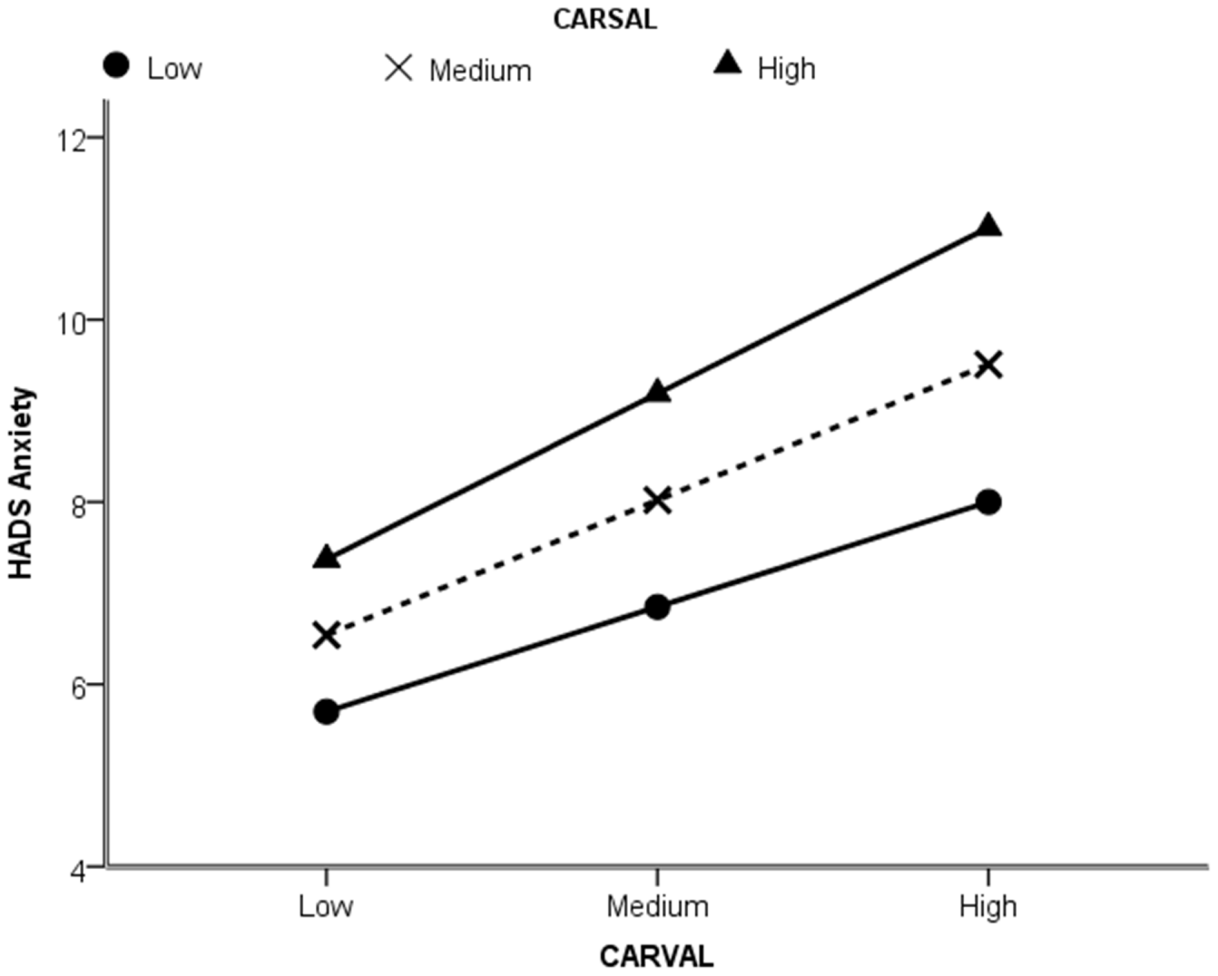
Moderation of CARVAL on HADS anxiety by CARSAL. Anxiety and appearance valence relationship, based on continuous data split by low appearance salience (lower line), moderate appearance salience and high appearance salience (upper line). Graph produced on basis of continuous data by ModGraph-I (19).

Similar patterns were observed for anxiety and depression. For anxiety, the interaction between CARVAL and CARSAL was (B = 0.08, *t*(1189) = 2.987, *p*<0.005). As can be seen in Figure 3, greater anxiety was associated with high negative valence and this was exacerbated by higher level of appearance salience. The moderation of CARVAL on HADS anxiety by CARSAL is shown in Figure 4.

**Figure 5:**
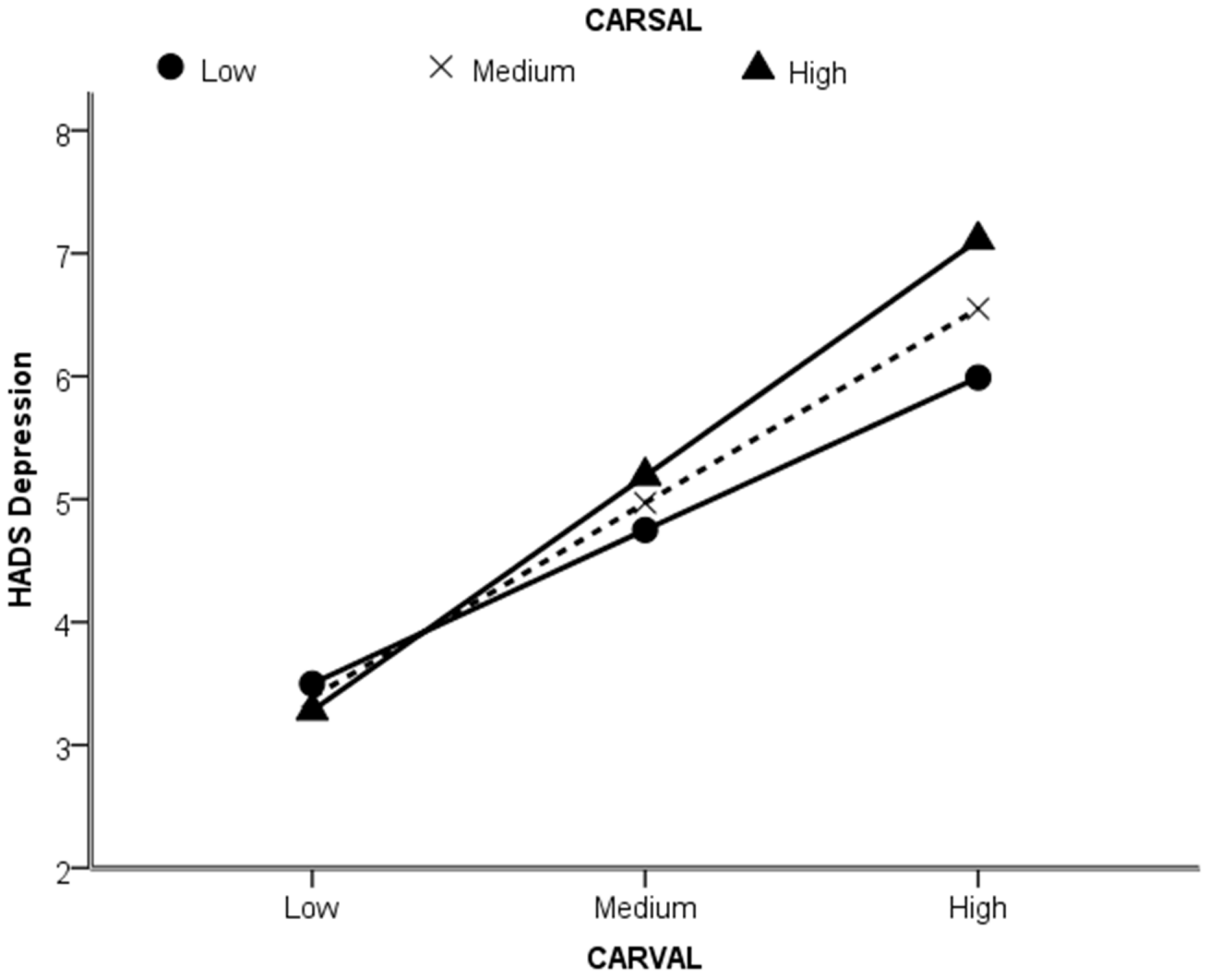
Moderation of CARVAL on HADS depression by CARSAL. Depression and appearance valence relationship, based on continuous data split by low appearance salience (lower line), moderate appearance salience and high appearance salience (upper line). Graph produced on basis of continuous data by ModGraph-I (19).

The predicted interaction between CARVAL and CARSAL was also observed in the depression scores, (B = 0.098, *t*(1189) = 3.625, *p*<0.001). As can be seen in Figure 5, higher depression scores were associated with high negative valence and this was exacerbated by higher level of appearance salience.

### Demographics

#### Gender differences

The mean salience score for men was 20.2 (*SD* = 6.72), lower than the mean score for women, 23.57 (*SD* = 5.41). This was significant *t* (542) = 8.30, *p*<.001.

Valence mean scores for men were 19.19 (*SD = *7.97), lower than the mean score for women, 22.50 (*SD* = 7.90). This was significant *t* (1180) = 6.54, *p*<.001. A descriptive comparison of total scores by gender between the current study, and [1] is shown in Table 3, demonstrating a similar pattern of mean scores.

**Table 3:** Mean and standard deviations of CARSAL and CARVAL across the current study and previous (general population) study [1], organised by gender.

Gender and study	Scale	N	Mean	Std.Deviation
Female,	CARSAL	844	23.6	5.4
current study	CARVAL	839	22.5	7.8
Male,	CARSAL	348	20.2	6.7
current study	CARVAL	343	19.2	8.0
Female,	CARSAL	429	23.6	4.8
previous study [1]	CARVAL	429	19.7	7.6
Male,	CARSAL	102	22.2	5.8
previous study [1]	CARVAL	102	16.4	6.8

#### Test–retest reliability

Pearson correlation indicated acceptable test-retest reliability for CARSAL (*r = *.70) and for CARVAL (*r = *.69).

## Discussion

Supplementary psychometric validation of the CARSAL and CARVAL measurement tool demonstrated that both measures are psychometrically sound for a population with a visible difference. It also demonstrated that there are similar underlying issues and relationships between constructs as seen in the general population. The analysis of the current and previous data also demonstrated that salience and valence are both conceptually independent and interdependent constructs. CARVAL is associated with appearance self-consciousness, anxiety and depression but further analysis confirmed that salience moderates the relation between valence and other psychosocial measures. The significantly higher scores for salience and valence of appearance for women are consistent with finding of the gender differences on levels of appearance concern and adjustment [3]. The brevity of the measures (six items for valence and five items for salience) may be of particular advantage when working with populations who may already be overburdened with other tests. Fewer items were used than in the original (non-clinical) analysis. However, the analysis reported here was conducted on a like-for-like basis with items from the original (non-clinical) data set, rather than total scores, enabling meaningful comparison to be made.

One of the limitations to this study was the use of a cross sectional design which did not allow for exploration of how salience and valence may fluctuate over time. Although the retest data provided validation of the reliability of the constructs, less is know about natural variation in appearance salience and appearance valence that may occur on a week-to-week, and month-to-month basis, particularly in relation to other relevant constructs such as anxiety and depression. However, with the validation of CARSAL and CARVAL in a clinical population, a tool is now available that would support investigation of this issue. Another limitation of the current study was that the scales under investigation were included as part of a battery of tests for a larger study examining appearance concerns. Although this conferred considerable advantage in terms of access to populations, and resulted in a well-powered sample, the drawback was that the salience of appearance concerns is likely to have been amplified for participants. This study did not attempt to measure sensitivity of the measures to change following interventions, and it would be beneficial to establish this in the future.

In conclusion, this study demonstrates that CARSAL and CARVAL are psychometrically valid, practical tools that can be used in both general and clinical populations.

## References

[1] Moss TP, Rosser BA (2012) The moderated relationship of appearance valence on appearance self consciousness: Development and testing of new measures of appearance schema components. PLoS One 7: e50605. 23226326 10.1371/journal.pone.0050605 PMC3511517

[2] Cash TF, Melnyk SE, Hrabosky JI (2004) The assessment of body image investment: An extensive revision of the appearance schemas inventory. Int J Eat Disord 35: 305–316. 15048946 10.1002/eat.10264

[3] Carr T, Moss T, Harris D (2005) The DAS24: A short form of the Derriford appearance scale DAS59 to measure individual responses to living with problems of appearance. British Journal of Health Psychology 10: 285–298. 15969855 10.1348/135910705X27613

[4] Djan R, Penington A (2013) A systematic review of questionnaires to measure the impact of appearance on quality of life for head and neck cancer patients. Journal of Plastic, Reconstructive & Aesthetic Surgery. 6: 627–637. 10.1016/j.bjps.2013.01.007 23394687

[5] Zigmond AS, Snaith R (1983) The Hospital Anxiety and Depression scale. Acta Psychiatr Scand 67: 361–370. 6880820 10.1111/j.1600-0447.1983.tb09716.x

[6] Comrey AL, Lee HB (1992) A first course in factor analysis. (2nd ed.). Hillsdale, NJ: Erlbaum.

[7] Clarke A, Thompson A, Rumsey N, Jenkinson E, Newell R (2013) CBT for Appearance Anxiety: Psychosocial Interventions for Anxiety Due to Visible Difference. John Wiley & Sons, Oxford.

[8] Bland JM, Altman DG (1997) *Statistics notes:* Cronbach’s alpha. British Medical Journal. 314: 572.

[9] Bjelland I, Dahl AA, Haug TT, Neckelmann D (2002) The validity of the Hospital Anxiety and Depression scale-an updated literature review. J Psychosom Res 52: 69–78. 11832252 10.1016/s0022-3999(01)00296-3

[10] Chatterjee S, Jamieson L, Wiseman F (1991) Identifying most influential observations in factor analysis, Marketing Science. 10: 145–160.

[11] Jackson DA (1993) Stopping rules in principal components analysis: a comparison of heuristical and statistical approaches. Ecology 74: 2204–2214.

[12] Velicer WF (1976) Determining the number of components from the matrix of partial correlations. Psychometrika 41: 321–327.

[13] Velicer WF, Eaton CA, Fava JL (2000) Construct explication through factor or component analysis: A review and evaluation of alternative procedures for determining the number of factors or components. 41–71 in RD Goffin and E Helmes, Eds., Problems and solutions in human assessment, Boston: Kluwer.

[14] Horn JL (1965) A rationale and test for the number of factors in factor analysis. Psychometrika 30: 179–185. 14306381 10.1007/BF02289447

[15] Cota AA, Longman RS, Holden RR, Fekken GC (1993) Comparing different methods for implementing parallel analysis: A practical index of accuracy, Educational and Psychological Measurement. 53: 865–876.

[16] Glorfeld LW (1995) An improvement on Horn’s parallel analysis methodology for selecting the correct number of factors to retain. Educational and Psychological Measurement 55: 377–393.

[17] Lorenzo-Seva U, ten Berge JMF (2006) Tucker’s congruence coefficient as a meaningful index of factor similarity, Methodology. 2: 57–64.

[18] Aiken LS, West SG (1991) Multiple regression: Testing and interpreting interactions. Sage Publications, Incorporated.

